# Knowledge of causes, clinical features and diagnosis of common zoonoses among medical practitioners in Tanzania

**DOI:** 10.1186/1471-2334-8-162

**Published:** 2008-12-02

**Authors:** Kunda John, Rudovic Kazwala, Godfrey S Mfinanga

**Affiliations:** 1 National Institute for Medical Research, Muhimbili Medical Research Centre, Dar es Salaam, Tanzania; 2 Sokoine University of Agriculture, Department of Veterinary Medicine, Morogoro, Tanzania

## Abstract

**Background:**

Many factors have been mentioned as contributing to under-diagnosis and under-reporting of zoonotic diseases particularly in the sub-Sahara African region. These include poor disease surveillance coverage, poor diagnostic capacity, the geographical distribution of those most affected and lack of clear strategies to address the plight of zoonotic diseases. The current study investigates the knowledge of medical practitioners of zoonotic diseases as a potential contributing factor to their under-diagnosis and hence under-reporting.

**Methods:**

The study was designed as a cross-sectional survey. Semi-structured open-ended questionnaire was administered to medical practitioners to establish the knowledge of anthrax, rabies, brucellosis, trypanosomiasis, echinococcosis and bovine tuberculosis in selected health facilities within urban and rural settings in Tanzania between April and May 2005. Frequency data were analyzed using likelihood ratio chi-square in Minitab version 14 to compare practitioners' knowledge of transmission, clinical features and diagnosis of the zoonoses in the two settings. For each analysis, likelihood ratio chi-square p-value of less than 0.05 was considered to be significant. Fisher's exact test was used where expected results were less than five.

**Results:**

Medical practitioners in rural health facilities had poor knowledge of transmission of sleeping sickness and clinical features of anthrax and rabies in humans compared to their urban counterparts. In both areas the practitioners had poor knowledge of how echinococcosis is transmitted to humans, clinical features of echinococcosis in humans, and diagnosis of bovine tuberculosis in humans.

**Conclusion:**

Knowledge of medical practitioners of zoonotic diseases could be a contributing factor to their under-diagnosis and under-reporting in Tanzania. Refresher courses on zoonotic diseases should be conducted particularly to practitioners in rural areas. More emphasis should be put on zoonotic diseases in teaching curricula of medical practitioners' training institutions in Tanzania to improve the diagnosis, reporting and control of zoonotic diseases. Veterinary and medical collaboration should be strengthened to enable more effective control of zoonotic diseases in Tanzania.

## Background

A total of 61% (n = 868) of all human diseases and 75% of emerging human pathogens are zoonotic [1]. Besides the fact that many emerging human diseases are zoonotic [2-5], its only now that they have been demonstrated by quantitative analysis as risk factors for disease emergence [1]. Both domestic and wild animals have been shown to be important reservoirs of zoonoses [6,7].

In Africa, bovine tuberculosis, brucellosis, anthrax, sleeping sickness, and rabies are still widespread [8,9] and in Tanzania, African trypanosomiasis, plague, rabies, brucellosis, anthrax and echinococcosis have been documented as being among the most common zoonoses [6]. In a study conducted in northern Tanzania in 2002, nineteen diseases were recorded as zoonoses by household members with rabies, tuberculosis, anthrax and brucellosis the top four zoonoses in pastoral, agro-pastoral and smallholder dairy farming systems [10]. Although the majority of households practiced at least one risk activity for transmission of zoonoses there was general lack of knowledge about the diseases [10,11].

Although human brucellosis is a notifiable disease in many countries, official figures do not fully reflect the number of people infected each year and the true incidence has been estimated to be between 10 and 25 times higher than what reported figures indicate [12]. Cases very often remain unrecognized and are thus treated as other diseases or as "fever of unknown origin" [12]. In Uganda, it was noted that despite the reported increase in the number of individuals infected with *Trypanosoma gambiense species *in the 1990s, WHO estimated that the figures represent only 10–15% of the actual number of infected individuals [13]. Poor referral systems, limited surveillance coverage, difficulty and delay in diagnosis by the health facilities have been contributing to the under-reporting of zoonoses. Patients on the other hand have been seeking alternative services such as those offered by traditional healers and hence delay to present to health facilities or failing to present at all making data on their diseases not available for epidemiological records [13-16].

Individuals as well as societies have been slow to act on zoonoses [17]. This could be due to insufficient systematic continuing education and opportunities to acquire new knowledge on zoonoses for those working in health institutions [18]. A physician attending to an ill veterinarian or a zookeeper will immediately suspect a wide array of diseases other than zoonoses; likewise a pediatrician attending to a sick child who recently received a puppy will not suspect an animal transmitted disease. All these underscores the fact that medical professionals have not been giving due consideration of animals as carriers of diseases that can be transmitted to humans [19]. This has resulted in poor quality of epidemiological data on zoonoses and their control measures on animal and human populations in particularly sub-Saharan Africa [20,21].

Translation of knowledge into proper care of patients is among the critical areas in health care delivery [22]. This is only possible if health service providers have the right knowledge of health problems they are dealing with. In some countries active continuing education programmes have been intensified to consolidate the knowledge of health workers [23]. Innovative educational approaches have also been addressed in target specific groups of health workers to facilitate the implementation of guideline-based recommendations in the management of patients [24]. Because targeted education is an integral part in improving the diagnosis of diseases [25], assessing the knowledge of practitioners could be an important step in identifying target receptors for public health education in Tanzania.

## Methods

### Types of medical practitioners involved

Diagnosis and treatment of patients in Tanzania is the responsibility of a range of health personnel including medical officers, assistant medical officers, and clinical officers (medical assistants). Medical officers have training to degree level while assistant medical officers and clinical officers do not have training to degree level. Assistant medical officer is taken as a higher category with more years of training than medical assistants. For the purpose of this study, all these categories of staff have been referred to as medical practitioners or practitioners.

### The study area

**Figure 1:**
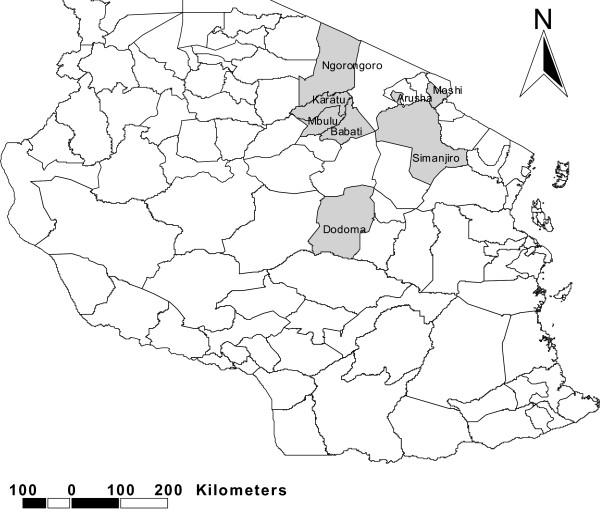
Map of Tanzania showing the study area.

The study was conducted in the districts of Ngorongoro, Karatu and Arusha in Arusha region, Mbulu, Babati and Simanjiro in Manyara region and Moshi in Kilimajaro regions in north-eastern Tanzania, and Dodoma urban in Dodoma region located in central Tanzania (Figure 1). Health facilities involved included Mount Meru, Karatu Lutheran and Endulen hospitals in Arusha region and Dareda missionary, Mbulu district, Babati district hospital and Simanjiro health centre in Manyara region. Others included Makole health centre and Dodoma regional hospital in Dodoma region and Mawenzi hospital in Kilimanjaro region. All the regions studied have the majority of people practicing animal husbandry [26]. Arusha and Manyara regions have subsistence farmers practicing both agro-pastoral and pastoral farming systems whereas farmers in Kilimanjaro and Dodoma regions practice mainly agro-pastoral system.

### Study design and sampling

A cross-sectional survey was conducted between April and May 2005. It focuses on areas in Tanzania containing a high proportion of livestock-keepers [26] within the central belt and northern Tanzania. Logistic and time constraints prevented access to regions in the southern highlands. A list of all medical facilities within the regions was compiled and assigned as urban or rural on the basis of Tanzania government regional administrative divisions. Four hospitals were selected at random from urban and six from rural communities within the six regions.

### Data collection

A semi-structured open-ended questionnaire was developed to assess knowledge of the causes, clinical features and diagnosis of anthrax, bovine tuberculosis, trypanosomiasis, rabies, echinococcosis and brucellosis. Field testing of the questionnaire was conducted in March 2005 at Wasso hospital in Ngorongoro district which was not included in the study. The focus of the questionnaire was on medical practitioners' knowledge considered important for identification and diagnosis of the zoonoses. On transmission, the emphasis was on knowledge of animal reservoirs and transmission routes and on clinical features, questions were asked about classical and pathognomonic features of zoonoses in humans and finally data were collected on knowledge of diagnostic protocols for zoonoses.

### Data analysis

All the responses were assessed in relation to the information provided by zoonoses text books [27,28] and were assigned as: "True" if the response was the same or closely similar to the documented, "False" if it was not and "Partial" if the respondent had some correct knowledge of a particular aspect of a zoonosis and incorrect on the other aspect. All the practitioners present on the first day of the visit and who agreed to participate with the study were enrolled. Two medical practitioners dropped out of the study citing time constraint. Time to fill in the questionnaire was allocated according to average time recorded during pre-testing (30 minutes). This was done to minimize sharing of knowledge and referring to text books that could have interfered with the analysis.

Medical practitioners were classified into levels of training as medical officers, assistant medical officers and clinical officers and then assigned into urban and rural areas according to the location of the health facilities. Frequency data were analyzed using likelihood ratio chi-square in Minitab version 14 (Minitab Inc. 2000, Release 14 for Windows, State College, Pennsylvania) to compare knowledge of transmission, clinical features and diagnosis of the zoonoses of the practitioners in the two settings. For each analysis, likelihood ratio chi-square p-value of less than 0.05 was considered to be significant. Fisher's exact test was used where expected results were less than 5.

Analysis was initially conducted in two phases. In the first phase responses from medical officers and assistant medical officers were combined and compared with responses from clinical officers. In this phase, two analyses were conducted. In the first analysis all the "Partial" responses were included as "True" responses and in the second analysis "Partial" responses were omitted and hence only "True" and "False" responses were included in the analysis. In the second phase, medical practitioners were divided into rural and urban and compared with respect to knowledge of different aspects of zoonoses. As above, analysis was conducted with "Partial" responses as "True" responses and repeated when omitted.

### Ethics

The study was cleared for ethics by the Medical Research Coordinating Committee of the National Institute for Medical Research in the Republic of Tanzania. Consent was also sought from all the health facilities and medical practitioners before being involved.

## Results

In total, four medical officers, six assistant medical officers and 27 clinical officers participated in the questionnaire (Table 1). Based on the location of the hospitals, seventeen medical practitioners were classified as working in rural hospitals and 20 in urban hospitals.

**Table 1:** Medical practitioners and health facilities involved with study

**Hospital**	**Assistant Medical Officers**	**Clinical Officers**	**Medical Officers**	**Total**
Babati		4		4

Dareda		2		2

Dodoma	2	2	2	6

Endulen	1	3		4

Karatu		2		2

Mawenzi		2	2	4

Makole		5		5

Mbulu	1	2		3

Mount Meru	2	3		5

Simanjiro		2		2

**Total**	**6**	**27**	**4**	**37**

When level of training was considered in the analysis, there was no significant difference between the two groups (medical officers combined with assistant medical officers compared to clinical officers) with respect to knowledge of zoonoses, showing that level of training was not a factor in determining the type of response. Analysis was therefore conducted basing on comparison of responses from medical practitioners in urban and rural hospitals with "Partial" knowledge responses omitted.

### Knowledge of practitioners of transmission of zoonoses

"True" responses with respect to transmission of rabies were recorded in a high proportion of practitioners in rural and urban areas (93.75%, 95% CI = 69.77–99.84, n = 19 and 94.74%, 95% CI = 73.97–99.86, n = 19 respectively) with no significant difference between them (Fisher's exact test, p = 1). Significantly more practitioners in urban hospitals appeared to have the correct knowledge of how sleeping sickness is transmitted compared to their rural counterparts (χ^2 ^= 4.2, df = 1, p < 0.05). In both urban and rural health facilities, only a few practitioners were observed to have the right knowledge of the transmission of echinococcosis (44.44%, 95% CI = 21.5–69.2, n = 18 and 23.53%, 95% CI = 6.8–49.8, n = 17 respectively), with no significant difference between urban and rural sites (χ^2 ^= 1.6, df = 1, p > 0.05) (Table 2).

**Table 2:** Knowledge of practitioners of transmission of common zoonoses

**Variable**	**Rural**				**Urban**				**Likelihood ratio/Fisher's exact test p-value**
	**No. gave correct response**	**%**	**No. gave wrong response**	**%**	**No. gave correct response**	**%**	**No. gave wrong response**	**%**	

Transmission anthrax	9	69.23	4	30.77	15	93.75	1	6.25	0.14*

Transmission brucellosis	12	92.31	1	7.69	10	66.67	5	33.33	0.17*

Transmission Rabies	15	93.75	1	6.25	18	94.74	1	5.26	1*

Transmission trypanosomiasis	7	46.67	8	53.33	16	80.00	4	20.00	0.04

Transmission bovine TB	11	84.62	2	15.38	15	88.24	2	11.76	1 *

Transmission echinococcosis	4	23.53	13	76.47	8	44.44	10	55.56	0.19

* Fisher's exact test p value

### Knowledge of practitioners of clinical features of zoonoses in humans

There was a significant difference between the knowledge of practitioners in the rural and urban hospitals on clinical features of anthrax and rabies. More practitioners in urban hospitals were found to have the correct knowledge of clinical features of anthrax and rabies compared to the practitioners in the rural hospitals (χ^2 ^= 4.6, df = 1, p < 0.05 and χ^2 ^= 6.991, df = 1, p-value < 0.01 respectively). In both urban and rural hospitals a few practitioners were observed to have the right knowledge of clinical features of echinococcosis (33.33%, 95% CI = 11.82–61.61, n = 15 and 47.06%, 95% CI = 22.98–72.2, n = 17 respectively), with no significant difference between them (χ^2 ^= 0.6, df = 1, p > 0.05) (Table 3).

**Table 3:** Knowledge of practitioners on clinical features of zoonoses

**Variable**	**Rural**				**Urban**				**Likelihood ratio/Fisher's exact test p-value**
	**No. gave correct response**	**%**	**No. gave wrong response**	**%**	**No. gave correct response**	**%**	**No. gave wrong response**	**%**	

Clinical features anthranx	5	35.71	9	64.29	12	75.00	4	25.00	0.03

Clinical features brucellosis	10	66.67	5	33.33	8	50.00	8	50.00	0.35

Clinical features rabies	7	43.75	9	56.25	17	85.00	3	15.00	0.008

Clinical features trypanosomiasis	11	61.11	7	38.89	11	73.33	4	26.67	0.46

Clinical features bovine TB	7	53.85	6	46.15	3	25.00	9	75.00	0.23*

Clinical features echinococcosis	5	33.33	10	66.67	8	47.06	9	52.94	0.43

* Fisher's exact test p value

### Knowledge of practitioners of diagnosis of zoonoses in humans

In both rural and urban hospitals, a few practitioners had the correct knowledge of type of samples and investigations to be conducted to rule out bovine tuberculosis (33.33%, 95% CI = 4.3–77.7, n = 6 and 47.06%, 95% CI = 22.9–72.2, n = 17 respectively) and the difference was not statistically significant (χ^2 ^= 1.8, d = 1, p > 0.05). There was no significant difference between the number of practitioners in rural and urban hospitals with correct knowledge of diagnosis of other zoonoses (Table 4).

**Table 4:** Knowledge of urban and rural practitioners on diagnosis of zoonoses

**Variable**	**Rural**				**Urban**				**Likelihood Ratio/Fisher's exact test p-value**
	**No. gave correct response**	**%**	**No. gave wrong response**	**%**	**No. gave correct response**	**%**	**No. gave wrong response**	**%**	

Diagnosis anthrax	7	43.75	9	56.25	10	58.82	7	41.18	0.38

Diagnosis brucellosis	13	86.67	2	13.33	9	56.25	7	43.75	0.11*

Diagnosis rabies	5	33.33	10	66.67	9	60.00	6	40.00	0.14

Diagnosis trypanosomiasis	12	75.00	4	25.00	16	84.21	3	15.79	0.68*

Diagnosis bovine TB	2	33.33	4	66.67	8	47.06	9	52.94	0.66*

Diagnosis echinococcosis	12	75.00	4	25.00	15	78.95	4	21.05	0.09

* Fisher's exact test p value

## Discussion

Diagnosis and hence reporting of diseases depend largely on the level of understanding of the diseases. Knowledge of reservoirs of zoonoses and the way they are transmitted to humans has enabled not only their diagnosis and reporting but also their control [29,30]. For instance, knowledge of animal reservoir and transmission modes has enabled the identification and control of zoonoses outbreaks in the world such as Rift Valley fever in Kenya and Somalia [31], Nipah virus in Malaysia and Singapore [32], West Nile virus in USA [33] and Hendra virus in Australia [34]. It has however been noted in some countries that zoonoses are causing prolonged and unnotified human suffering [19]. Where malaria is endemic, diseases such as brucellosis and anthrax have been under-diagnosed because of their similarities in clinical presentations [12]. It is therefore important to optimize the diagnosis of other diseases such as zoonoses that have significant socio-economic impact on human life.

According to the population and housing census conducted in Tanzania in 2002, over 75% of the Tanzanian population is in rural areas and keep more livestock compared to their urban counterparts [26,35]. One would therefore expect more awareness of zoonoses in rural than in urban areas. In the current study however, it was found that practitioners in the rural areas had poor knowledge of how sleeping sickness is transmitted to humans and clinical features of anthrax and rabies. In both areas the practitioners had poor knowledge of how echinococcosis is transmited, how it presents, and how to go about investigating bovine tuberculosis in humans. Inadequate knowledge of any aspect of a disease is a potential contributing factor to misdiagnosis. For instance, if a practitioner is not well informed of how the diseases manifests or does not know how to investigate for its presence, there is a higher chance of misdiagnosis as one would not know which disease to investigate and how.

Adequate knowledge of animal reservoirs and transmission routes enable practitioners to focus on key areas related to the disease and hence reach the definitive diagnosis easier and earlier enough for prompt management of the disease [36]. The results of the study indicate that it is possible some zoonoses are missed by those entrusted with the duty of identifying them. Many reasons could explain these findings. These include practitioners concentrating on endemic diseases or diseases that have been common in their areas and ignore zoonoses that are increasingly becoming of public health importance and the possibility that teaching curricula in medical training institutions do no put due emphasis on zoonoses. The location of practitioners in the distant underprivileged rural areas could also explain the differences in the level of knowledge. Refresher programmes, seminars and workshops are more convenient and cheaper to conduct amongst staff in urban than in rural areas.

In Tanzania, like in other sub-Sahara African countries, there is a poor diagnostic capacity of many diseases including zoonoses. Laboratories particularly in the rural areas where there is the majority of the population are poorly equipped and can not diagnose most of the emerging and re-emerging diseases. Besides this fact, increased awareness of diseases among health workers and the community is still the most important area in disease control. Before more weight is put on increasing the diagnostic capacity of diseases, efforts should be made to equip the practitioners and the general community with adequate knowledge of zoonoses.

The study has shed some light on an area that has not been thought before as a possible contributing factor to under-reporting of diseases such as zoonoses. A larger study covering a wider area using random sampling could provide more conclusive results. In the current study, the prevailing weather condition necessitated a convenient sampling which could not be the best method to conduct such a study. The distribution of practitioners as presented in the study represents the distribution of health staff in Tanzania. The majority of senior health staff are found in urban areas of big cities where patients from rural facilities are referred for expertise management. However, it was established in the study that there was no significant difference of level of knowledge of zoonoses amongst different levels of practitioners.

## Conclusion

Knowledge of medical practitioners of zoonoses could be among the contributing factors to under-diagnosis and under-reporting of zoonoses in Tanzania. There is a need for refresher programmes amongst the practitioners but also their teaching curricula should put more emphasis on zoonoses. This will provide practitioners with adequate knowledge of zoonoses prevention and control in the community. The study suggests that any intervention to be undertaken should give priority to practitioners in the rural areas.

Collaboration between veterinary and medical personnel should also be strengthened in Tanzania. This should include sharing knowledge on zoonoses and working together to identify and control zoonoses which are increasingly becoming diseases of public health importance in Tanzania.

## Competing interests

The authors declare that they have no competing interests.

## Authors' contributions

KJ, RK and GM were involved in the design of the study, KJ supervised field work, KJ was involved in data analysis and KJ, RK and GM were involved in manuscript write-up. All authors have read and approved the final manuscript.

## Pre-publication history

The pre-publication history for this paper can be accessed here:


